# Analysis of emerging trends and hot spots in respiratory biomechanics from 2003 to 2022 based on CiteSpace

**DOI:** 10.3389/fphys.2023.1190155

**Published:** 2023-07-20

**Authors:** Xiaofei Huang, Jiaqi Zheng, Ye Ma, Meijin Hou, Xiangbin Wang

**Affiliations:** ^1^ College of Rehabilitation Medicine, Fujian University of Traditional Chinese Medicine, Fuzhou, China; ^2^ Key Laboratory of Orthopedics & Traumatology of Traditional Chinese Medicine and Rehabilitation Ministry of Education, Fujian University of Traditional Chinese Medicine, Fuzhou, China; ^3^ Research Academy of Grand Health, Faculty of Sports Sciences, Ningbo University, Ningbo, China; ^4^ National-Local Joint Engineering Research Center of Rehabilitation Medicine Technology, Fuzhou, China

**Keywords:** respiratory biomechanics, CiteSpace, bibliometric analysis, radiotherapy, organ motion, sensor

## Abstract

**Introduction:** With the global prevalence of coronavirus disease 2019 (COVID-19), an increasing number of people are experiencing respiratory discomfort. Respiratory biomechanics can monitor breathing patterns and respiratory movements and it is easier to prevent, diagnose, treat or rehabilitate. However, there is still a lack of global knowledge structure in the field of respiratory biomechanics. With the help of CiteSpace software, we aim to help researchers identify potential collaborators and collaborating institutions, hotspots and research frontiers in respiratory biomechanics.

**Methods:** Articles on respiratory biomechanics from 2003 to 2022 were retrieved from the Web of Science Core Collection by using a specific strategy, resulting a total of 2,850 publications. We used CiteSpace 6.1.R6 to analyze the year of publication, journal/journals cited, country, institution, author/authors cited, references, keywords and research trends. Co-citation maps were created to visually observe research hot spots and knowledge structures.

**Results and discussion:** The number of annual publications gradually increased over the past 20 years. Medical Physics published the most articles and had the most citations in this study. The United States was the most influential country, with the highest number and centrality of publications. The most productive and influential institution was Harvard University in the United States. Keall PJ was the most productive author and MCCLELLAND JR was the most cited authors The article by Keall PJ (2006) article (cocitation counts: 55) and the article by McClelland JR (2013) were the most representative and symbolic references, with the highest cocitation number and centrality, respectively. The top keywords were “radiotherapy”, “volume”, and “ventilation”. The top Frontier keywords were “organ motion,” “deep inspiration,” and “deep learning”. The keywords were clustered to form seven labels. Currently, the main area of research in respiratory biomechanics is respiratory motion related to imaging techniques. Future research may focus on respiratory assistance techniques and respiratory detection techniques. At the same time, in the future, we will pay attention to personalized medicine and precision medicine, so that people can monitor their health status anytime and anywhere.

## 1 Introduction

Respiration is the process of gas exchange between the body and the external environment and involves three links: external respiration (lung ventilation and lung gas exchange), gas transport in the blood, and internal respiration (gas exchange between tissue cells and blood). There are two modes of normal breathing: abdominal breathing and thoracic breathing. The abdominal breathing is defined as when the contraction and relaxation of the diaphragm causes displacement of the organs in the abdominal cavity to shift, causing the abdomen to fluctuate. The thoracic breathing is defined as the when respiratory movement is mainly the movement of the intercostal muscles, the activity of the diaphragm is weak, and the chest expansion is obvious during breathing. Respiratory-related symptoms may include abnormal respiratory rate, abnormal respiratory rhythm, changes in respiratory status, and the like. Abnormal respiratory rate refers to an increase or decrease in the number of breaths. A normal adult’s respiratory rate is usually between 12 and 20 breaths per minute. Abnormal respiratory rhythm refers to irregular breathing rhythm or abnormal changes, such as rapid superficial breathing, apnea, etc. Changes in respiratory status refer to changes in respiratory status such as excessive breathing effort, dyspnea, superficial breathing, hoarseness, and shortness of breath.

Respiratory biomechanics is the studies of the forces acting on the human respiratory system and the responses produced by breathing (Yentes et al., 2022). The respiratory system itself is mechanical, therefore it is best to use biomechanical research methods to study respiratory movements. Based on this, biomechanical methods can be used to quantify the chest and abdominal movements generated by respiration and thus reflect respiratory function. In addition, biomechanics can also evaluate mechanical ventilation and fluid dynamic and comprehensively detect abnormal breathing patterns and respiratory movements in clinical practice. It then gradually forms a branch of biomechanics, respiratory biomechanics. In order to evaluate respiratory status and monitor respiratory-related indicators, modern medical technology provides a variety of sensor detection techniques combined with respiratory biomechanics, which can monitor respiratory-related indicators more accurately. Such as the respiratory belts, the sensor band can be fixed to the patient’s chest or abdomen to monitor respiratory frequency and rhythm by measuring chest and abdominal movement. The peak flow meters device is commonly used to evaluate respiratory function in patients with asthma. Patients need to blow into the flowmeter and assess their breathing status by measuring the intensity and speed of the air flow. Wireless chest straps, a sensor band that can be attached to the chest, assesses respiratory status by measuring heart rate, respiratory rate, and body movement. Sleep monitoring devices, which is usually used to assess sleep disorders, can monitor breathing by measuring respiratory rate, apnea, blood oxygen levels, and so on. There are also gas sensors that detect ppt-level nitrogen dioxide (NO_2_) at room temperature (Yuan et al., 2022). By using this highly sensitive and selective gas sensor, the concentration of NO_2_ in breathing air can be monitored and detected in real time. This helps to identify and assess air pollution problems related to respiratory health, and take corresponding measures to reduce potential hazards.

There are also new developments in materials. Polypyrrole (Zang et al., 2022) is a kind of polymer material with conductive properties. Under certain conditions, the transport behavior of polypyrrole is similar to that of metal, with the characteristics of high conductivity and free electrons. In the field of medicine, the metal-like transport properties of polypyrrole can be used to develop highly sensitive and adjustable biosensors to monitor biomolecules, biological signals or physiological parameters. In the field of respiration, it can be used to develop new electrode materials, which can be used in respiratory instruments or respiratory therapy equipment. This material has excellent electrical conductivity and controllability, which can improve the performance and adaptability of the equipment, and provide a more effective solution for respiratory therapy. What’s more, there is new research (Kamalabadi et al., 2022) on gas sensor based on polypyrrole/silver nanoparticle film, which is used to detect ammonia in exhaled gas. The ammonia in the exhaled air can provide important information about the condition of the lungs and respiratory system. The sensor is designed and prepared to provide a reliable, sensitive and selective detection method for rapid diagnosis and monitoring of respiratory-related diseases, such as COVID-19 patients with acute renal injury. Through this sensor, the information of ammonia in exhaled air can be obtained non-invasively, which is helpful to diagnose and monitor the development and therapeutic effect of respiratory system-related diseases. In addition, the design and preparation method of the sensor provides a reference for the development of other respiratory-related gas sensors.

In additions, nanogenerator (Long et al., 2021) is a kind of device that can convert mechanical energy into electrical energy, which uses the characteristics of nanomaterials to collect and convert energy. It has some potential applications in the field of respiratory monitoring, nanogenerator technology to develop devices that can collect energy from respiratory movements. Because breathing is one of the natural movements of the human body, when we breathe, the chest and abdomen will expand and contract. This mechanical movement can be converted into electricity through an appropriate mechanism and can be used to power respiratory monitoring equipment. By integrating Nanogenerator into breathing belts, chest sensors or other wearable devices, small amounts of energy generated by breathing can be collected and converted into electrical power devices. This avoids or reduces reliance on traditional batteries, extends the life of the device, and provides a more portable and self-sufficient solution.

Researchers have established respiratory mechanics models, which are mainly classified into first-order linear models and respiratory control models. The most typical and simplistic among them is a first-order linear model, which treats the respiratory system as a container with a single degree of freedom in a three-dimensional space. The mechanical model of the respiratory system depicts the mechanical process and mechanism of lung ventilation. The establishment of respiratory movement model enables the identification of abnormal patterns, to help clinical diagnosis of diseases, while using mechanical ventilation technology and fluid dynamics intervention treatment, such as COPD, obstructive sleep apnea hypoventilation syndrome, pneumothorax, etc. The worldwide spread of COVID-19 has led to an increase in morbidity and mortality worldwide, bringing heavy social and economic burdens (Fajnzylber et al., 2020). At the same time, the long-term health sequelae of COVID-19 are an important public health issue. Increasing evidence (Blomberg et al., 2021; Groff et al., 2021; Huang et al., 2021) suggested that dyspnea, cough, and fatigue are common sequelae of severe acute respiratory syndrome Coronavirus 2 (SARS-CoV-2) infection. Currently, in the treatment of 2019 novel coronavirus (2019-nCoV) infected pneumonia (Jin et al., 2020), severe and critically ill patients require respiratory support, such as nasal cannula or mask oxygen, nasal high-flow oxygen therapy or noninvasive ventilation, invasive mechanical ventilation, airway management, and extracorporeal membrane oxygenation (ECMO). Respiratory biomechanics not only provides theoretical support for the design of new medical devices, but also provides better treatment recommendations for patients and predicts public health. Therefore, more and more researchers have devoted themselves to the field of respiratory biomechanics, including observation, diagnosis, monitoring and rehabilitation. It has a wide range of applications and is highly beneficial to society. Respiratory biomechanics is an increasingly important field nowadays, but few studies have focused on its comprehensive knowledge structure.

At present, the application of respiratory biomechanics has been reviewed from several perspectives. Neelakantan et al. (2022) described the biomechanical characteristics of the lung and reviewed a series of lung model paradigms based on physiological and imaging data to facilitate individualized diagnosis, prognosis and therapeutic evaluation of respiratory diseases. Another review (McClelland et al., 2013) provided an overview of the use of respiratory motion models and algorithms, suggesting possibilities for applications in various fields and laying the groundwork for later developments in the field. Additionally, a recent review (Karbing et al., 2022) of new developments in the field of respiratory monitoring over the last year found that many involved the application of monitoring for mechanical ventilation, especially related to intensive care. The review also put forward some suggestions to improve the existing measurement techniques, guide the decision of mechanical ventilation and explore new ventilation modes. What’s more, Duan et al. (2021) summarized the latest development of humidity sensors in the field of human-related humidity detection. This paper summarizes the latest progress of humidity sensors in the field of human-related humidity detection, which provides a new possibility to improve the accuracy and convenience of health monitoring and medical diagnosis. In addition, the article also introduces some innovative applications in the detection of human humidity. These applications include respiratory monitoring, skin humidity monitoring, sweat analysis and exercise monitoring. These applications make humidity sensors can be used in disease diagnosis, exercise physiology research, sleep monitoring and intelligent health monitoring and other fields.

Bibliometrics refers to the quantitative analysis of all knowledge vectors published through mathematical and statistical methods. It can assess the impact of authors, institutions, countries and keywords on the development of a particular field based on the number of publications and the frequency of citations, which helps to find trends, hotspots and form knowledge structures (Roldan-Valadez et al., 2019; Zheng et al., 2022). However, there is a lack of visual analysis of respiratory biomechanics trends, key authors, and research hotspots from a bibliometric perspective. Bibliometric research can complement the field of respiratory biomechanics by providing new insights for further development. CiteSpace is a common bibliometric analysis tool, which can provide visual information and potential research directions for researchers with the support of computational and visual analysis methods (Chen and Song, 2019). Therefore, this study aims to use CiteSpace for visual analysis to identify the hot areas of respiratory biomechanics research. By doing so, researchers can better understand the research trend and current situation of respiratory biomechanics and guide future research.

## 2 Materials and methods

### 2.1 Data acquisition

The data of this study were collected from the Web of Science Core Collection in January 2023. The data search strategy was as follows: Topic= (respiration or breath*) and Topic= (Biomechanic* or motion analysis). We set restrictions on the retrieval period time and showed the results for 2003 to 2022. With no restrictions on language and literature type, we obtained 3,927 records. We then processed the retrieved records and obtained 2,850 records according to the following criteria.

#### 2.1.1 Inclusion criteria

The inclusion criteria were articles, proceedings papers, and reviews on respiratory biomechanics, that were formally published, had comprehensive research data, and not including animal mechanics.

#### 2.1.2 Exclusion criteria

The exclusion criteria were (Yentes et al., 2022): articles collected by hand and telephone (Yuan et al., 2022); articles not officially published; (Zang et al., 2022); the document type of letter, meeting abstract, editorial material, etc. (Kamalabadi et al., 2022) repeated publications (Long et al., 2021); unrelated articles.

#### 2.1.3 Quality assessment

English articles that met the inclusion criteria were included in the analysis.

### 2.2 CiteSpace

CiteSpace is an information visualization software developed by Professor Chaomei Chen that uses the Java language to analyze the evolution of disciplines and Frontier issues by drawing a series of visual knowledge maps (Chen and Chen, 2005). The size of the node indicates the frequency or number of occurrences of the reference, and its color represents the year of publication. Nodes with high centrality (>0.1) are usually considered hotspots or turning points in the field, with the purple outer ring of the node representing centrality. Additionally, CiteSpace provides two metrics, modularity Q (*Q* value) and mean silhouette (*S* value), based on the clarity of network structure and clustering. When the *Q* value >0.3, the cluster structure is significant and when the S value reaches 0.7, the clustering is considered convincing. The version used in this research was 6.1.R6 (64-bit). We used CiteSpace with the following parameters: time slicing (2003–2022), years per slice (Yentes et al., 2022), term source (all selections), node type (one at a time), selection criteria (top 50 objects), pruning (pruning sliced networks), and visualization (cluster view-static). In this study, CiteSpace provided analysis on researching journal/cited journals, country, institution, author/cited authors, references, keywords and research trends.

## 3 Results

### 3.1 Analysis of annual publications

**FIGURE 1:**
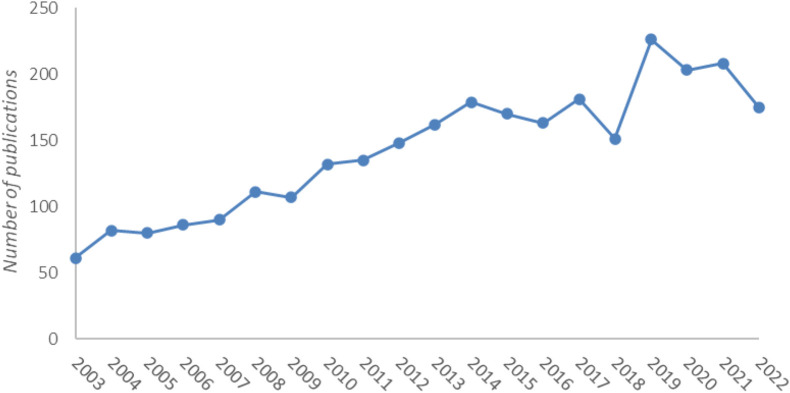
Annual number of published articles from 2003 to 2022.

A total of 2,850 records were obtained. As shown Figure 1, the number of publications has steadily increased with some fluctuations over 20 years. The study of respiratory mechanics began in the 20th century. In the 1960s, Jodat R.W. (Jodat et al., 1966). proposed a mechanical model of respiratory mechanics. However, Peters R.M. ‘s (Hilberman et al., 1969; Hilberman et al., 1972) mechanical model of the respiratory system was not based on anatomy, but on the respiratory process, and a phase method was proposed to calculate the respiratory mechanics using digital computer. The period from 2003 to 2014 showed sustained growth, with the number of publications exceeding 100 in 2008, indicating that the field received considerable attention in the 2010s. There was a significant increase from 2018 to 2019, with 226 records in 2019, which may be related to the impact of the COVID-19. The number of record in 2022 is 175, indicating a drop in activity. However, in the age of a respiratory pandemic, there is no doubt that more attention has been paid to the problems caused by respiratory diseases and advancing our understanding of respiratory biomechanics is more pressing than ever before. Therefore, it is likely to continue to thrive in the future according to the above analysis.

### 3.2 Analysis of journals and cited journals

Table 1 demonstrates the top five journals by the number of publications, most of which are related to medical physics. According to the most recent Journal Citation Report, their average impact factor was 4.5346.

**TABLE 1:** Top five most productive journals related to respiratory biomechanics.

Rank	Publications	Journal/IF^a^
1	263	Medical physics/4.506
2	184	Magnetic resonancein medicine/3.737
3	183	Physicsin medicine and biology/4.174
4	110	International journal of radiation oncology biology physics/8.013
5	70	Journal of applied clinical medical physics/2.243

^a^ IF, impact factor; IF, in category according to Journal Citation Reports (2021).

**FIGURE 2:**
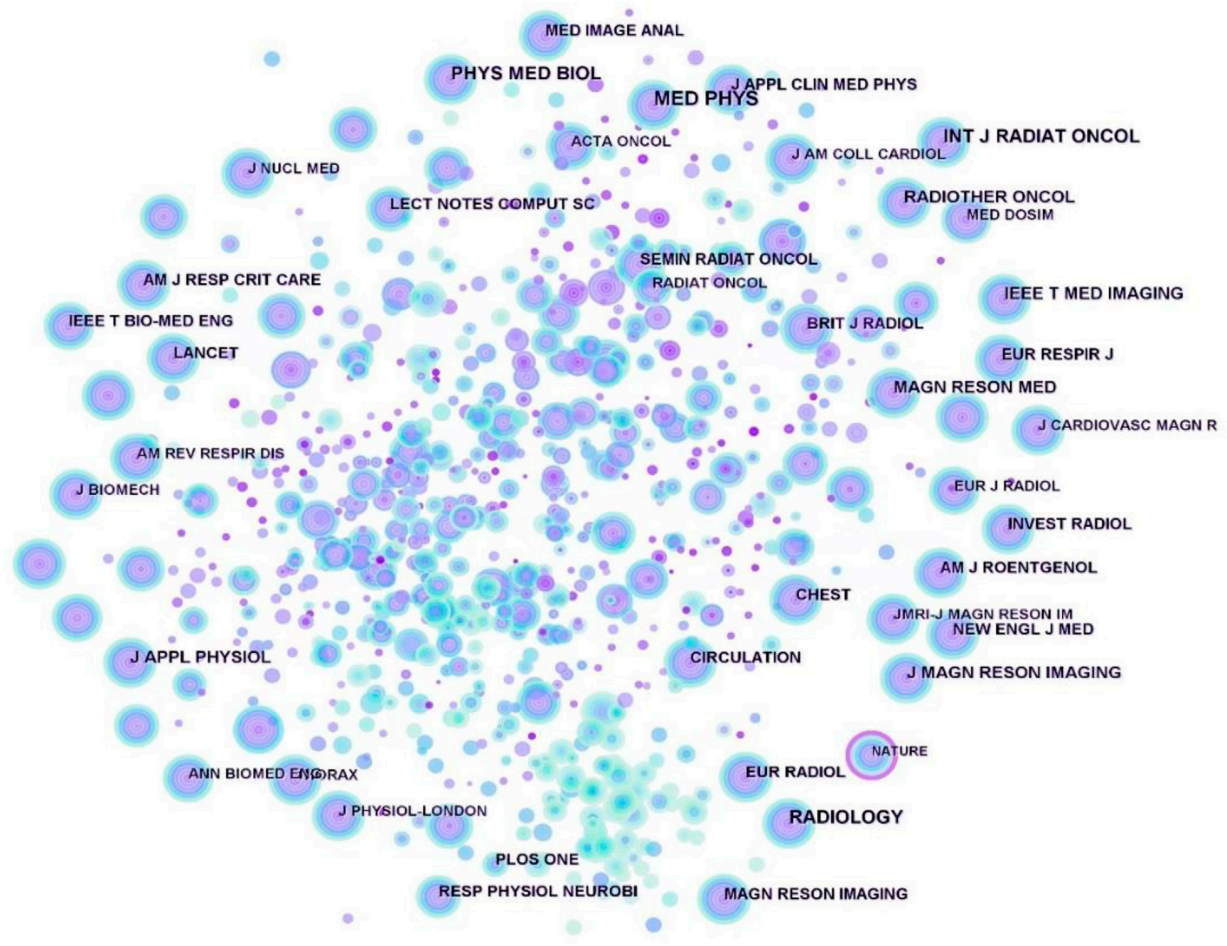
Map of cited journals for respiratory biomechanics from 2003 to 2022.

In addition, a cited journals map was generated by CiteSpace (Figure 2), resulting in 815 nodes and 7,066 links. The nodes in the map represent journals and these nodes have almost no purple rings, indicating that the centrality of these journals is not high, with the highest being 0.12 for Nature. Table 2 shows the top 10 cited journals related to respiratory biomechanics. We then analyzed the bibliometric information of these ten journals in Table 3. Since the five selected bibliometrics depicted non-normal distributions, we reported the median, quartiles and interquartile range (IQR). Most of the cited journals belonged to Q1, and half of them had impact factor above 6.01. The average IF, Eigenfactor, CiteScore, SNIP, and SJR of cited journals were 6.01, 0.0395, 9.2, 1.777, and 1.704, respectively.

**TABLE 2:** Top 10 cited journals related to respiratory biomechanics.

Rank	Frequency	Cited journal
1	1108	Medical physics
2	977	Physics in medicine and biology
3	912	International journal of radiation oncology biology physics
4	756	Radiology
5	650	Journal of applied physiology
6	625	Magnetic resonance in medicine
7	619	Radiotherapy and oncology
8	602	Journal of magnetic resonance imaging
9	573	IEEE transactions on medical imaging
10	390	American journal of respiratory and critical care medicine

**TABLE 3:** Descriptive statistics of the bibliometrics from the top-10 cited journals.

Bibliometrics	Median	25	75	IQR
IF	6.01	4.257	10.281	6.024
Eigenfactor	0.0395	0.03325	0.062	0.02875
CiteScore	9.2	6.875	16.6	9.725
SNIP	1.777	1.391	3.41575	2.02475
SJR	1.704	1.2345	3.52025	2.28575

### 3.3 Analysis of countries

**FIGURE 3:**
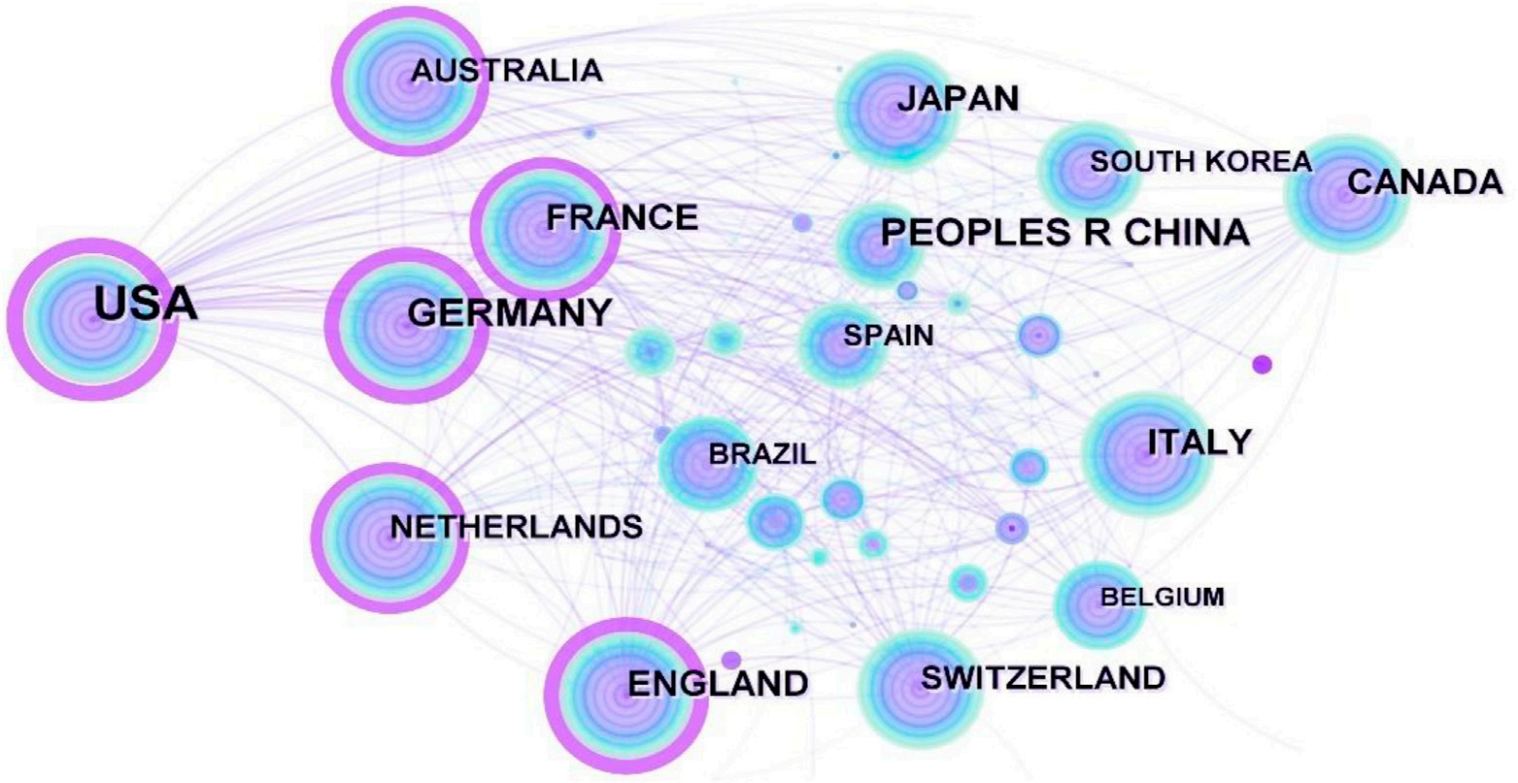
Map of countries researching respiratory biomechanics from 2003 to 2022.

The data on geographical distribution (Figure 3) were from CiteSpace. Research on respiratory biomechanics was conducted in 73 countries. The top five countries for publication are shown in Table 4. The United States was the leading contributor, accounting for 1,124 of the 2,850 total article (approximately one-third). Germany and China ranked in the second and third positions, while other countries remained below. In addition, China was the most productive country in Asia with 258 articles. In recent years, China has focused on wearable devices, which have potential for remote monitoring and improving access to healthcare. For example, a study (Xu et al., 2021) proposed a wireless, portable pulmonary function monitor for rehabilitation care after COVID-19. The practical design, which utilized wireless technology and a portable device, presented a low-cost and convenient method for pulmonary function monitoring during rehabilitation from COVID-19.

**TABLE 4:** Top 5 countries in number of publications related to respiratory biomechanics.

Rank	Publications	Countries	Years
1	1,124	United States	2003
2	369	Germany	2003
3	258	Peoples R china	2003
4	240	England	2003
5	167	France	2004

Figure 3 shows that the purple ring represented the centrality, with the United States (0.51), England (0.25), Germany (0.23), France (0.15), and Netherlands (0.13) being the top five countries in terms of centrality. The United States had a notable advantage in terms of quantity and importance. Furthermore, it is evident that research in respiratory biomechanics is predominantly conducted in developed countries. However, recent years, there has been a significant increase in the number of articles originating from Asian countries such as China, and South Korea, indicating a growing interest in respiratory biomechanics in these regions.

### 3.4 Distribution of institutions

**FIGURE 4:**
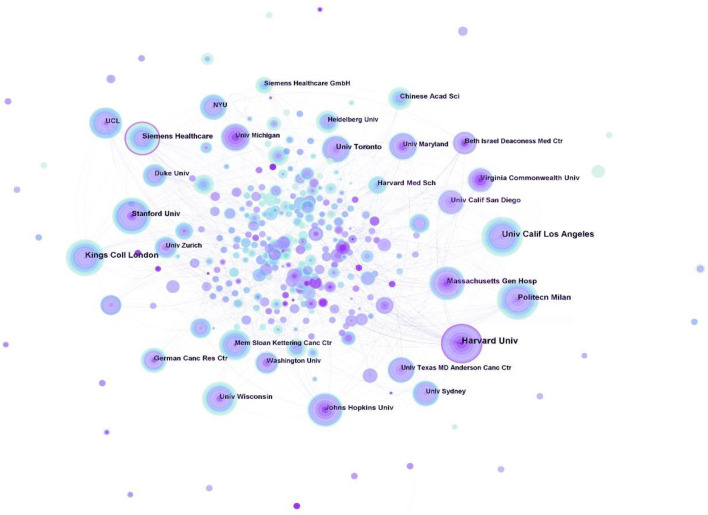
Map of institutions researching respiratory biomechanics from 2003 to 2022.

In Figure 4, the top 5 institutions dedicated to the field of respiratory biomechanics were all universities, including Harvard University, University California Los Angeles, King’s College London, Politecnico di Milano, and Stanford University. Moreover, the top 5 institutions in terms of centrality were Harvard University (0.12), Siemens Healthcare (0.11), Kings Coll London (0.09), Harvard Med Sch (0.08), and Stanford University (0.07).

The analysis of publications and centrality demonstrated that the primary research institutions were Harvard University (United States) and King’s College London (United Kingdom). In its early years, Harvard University primarily focused more on respiratory gating technique, but in recent years, it has shifted its focus towards instrumentation development. King’s College London’s research on respiratory biomechanics encompasses imaging, respiratory motion, among other areas.

### 3.5 Analysis of authors and cited authors

**FIGURE 5:**
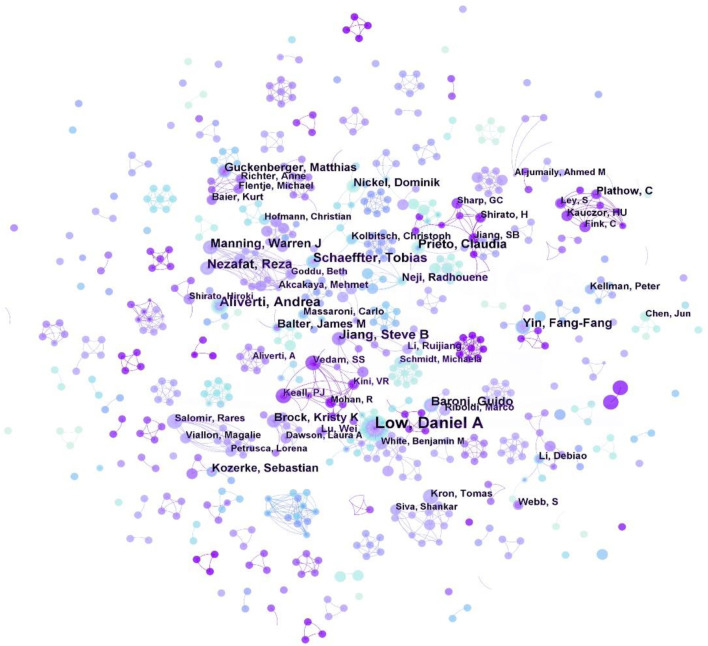
Map of authors related to respiratory biomechanics from 2003 to 2022.

The CiteSpace-generated author map presented in Figure 5 consist of 794 nodes and 1,231 links, with each node representing an authors. Collaborative groups formed connections with one another, and there were also individual authors scattered throughout the map. The top five authors in Figure 5 were Keall PJ (33), DANIEL LOW (Seppenwoolde et al., 2002), Balter JM (Hilberman et al., 1972), Baroni, Guido (Hilberman et al., 1972), and Jiang SB (Hilberman et al., 1969).

In a collaborative effort, DANIEL LOW, Keall et al. (2006) reported on the management of respiratory motion in radiation oncology. Additionally, Keall PJ, and Balter JM (Ruan et al., 2009a; Ruan et al., 2009b) analyzed the characteristics of various aspects of real-time profiling of respiratory motion, and developed a system for predicting the location of abdominal or thoracic tumors based on baseline drift, frequency changes, and fundamental pattern changes.

**FIGURE 6:**
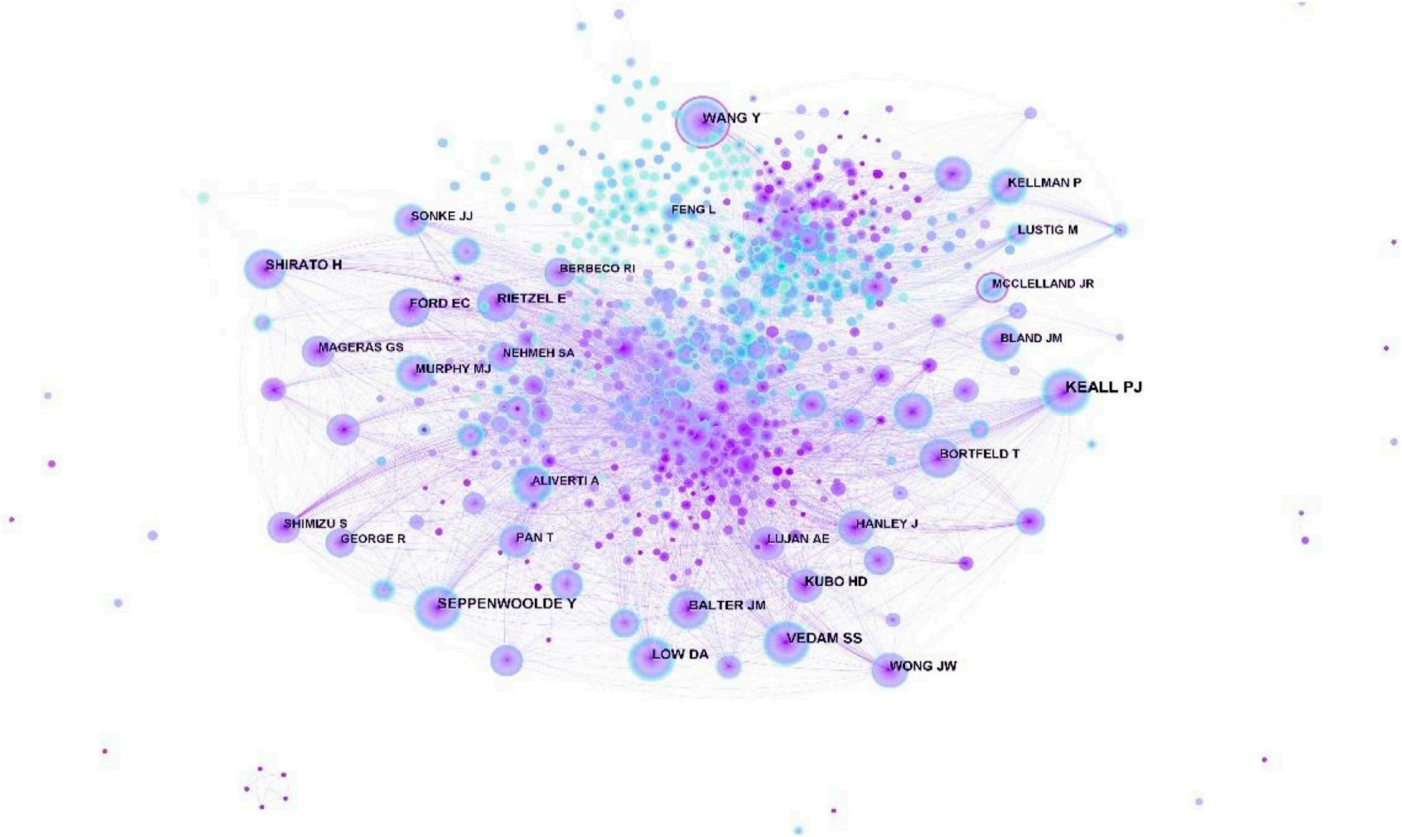
Map of cited authors related to respiratory biomechanics from 2003 to 2022.

The map of cited authors is shown in Figure 6. Table 5, KEALL PJ had the highest number of citations, followed by VEDAM SS, SEPPENWOOLDE Y, SHIRATO H, and LOW D. The top five authors in terms of centrality were WANG Y, MCCLELLAND JR, PLATHOW C, KEALL PJ and RUECKERT D. In Figure 6, we can observe that the node of “WANG Y” and “MCCLELLAND JR” have obvious purple rings, indicating a high mediating effect. Wang et al. (2013) works in the United States and focuses on nuclear medicine and medical imaging and radiology, among others, using machine learning algorithms. MCCLELLAND JR has worked at University College London, where he focused on imaging-based respiratory motion (Manber et al., 2016; Hanson et al., 2021).

**TABLE 5:** Top five Frequency and centrality of cited authors related to respiratory biomechanics.

Rank	Frequency	Author	Rank	Centrality	Author
1	396	Keall PJ	1	0.14	Wang Y
3	225	Vedam SS	2	0.14	Mcclelland JR
2	237	Seppenwoolde Y	3	0.1	Plathow C
4	184	Shirato H	5	0.09	Keall PJ
5	173	Low D	4	0.09	Rueckert D

Combining authors, cited authors, and centrality, Keall PJ was a professor in the field and had a major impact on the development of respiratory biomechanics. He has worked at the University of Sydney, focusing on imaging-based respiratory motion and in particular 4-dimensional computed tomography (4D-CT). In conventional CT examination, the motion error caused by respiratory motion was ignored, which reduced the accuracy of target delineation. As a result, the dose calculation in the final radiotherapy process was not accurate, which brought more uncertainties to radiotherapy. In order to accurately capture the motion trajectory of tumors, 4D CT technology was born. For 4D-CT scanning, the CT machine needs a partner-respiratory gating system, which means it needs to detect respiratory signals. In the positioning step, the patient’s respiratory curve should be collected during CT scan to reconstruct the image information of each phase, namely, 4D image reconstruction. One of his points was that the simultaneous use of audiovisual biofeedback (AVB) and breath gating could effectively improve 4D-CT interventions (Pollock et al., 2016).

### 3.6 Analysis of cited references

**FIGURE 7:**
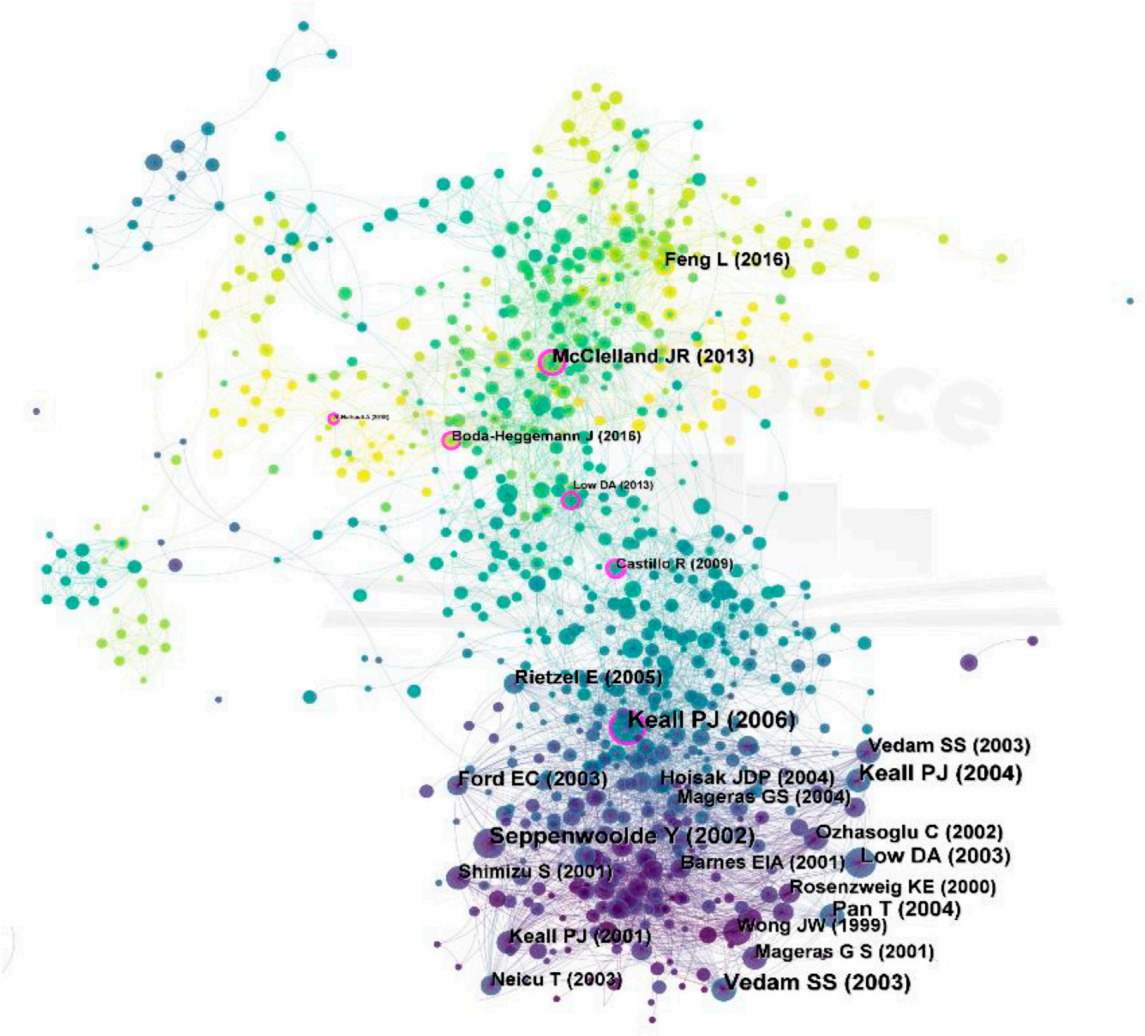
Map of cited references to respiratory biomechanics from 2003 to 2022.

A map of cited references is shown in Figure 7. In terms of citation frequency, the first article was published by Keall PJ in 2006 (23). The article described the issues and approaches caused by respiratory motion during therapeutic radiation and provided recommendations for respiratory motion management. Patient respiratory motion can result in a large error in the precise radiotherapy of thoracic and abdominal tumors. In the past, the delineation of the target volume for thoracic and abdominal tumors was based on clinical experience. In order to avoid missing the target volume, a larger margin was used, which unnecessarily irradiated a large area of normal organs. Seppenwoolde Y’s article in 2002 also addressed related issues and ranked second (Seppenwoolde et al., 2002). In their work, they measured tumor motion in three directions during radiotherapy, accounting for differences in respiratory levels during treatment, and concluded that accurate respiratory gating or CT scans during radiotherapy were necessary. The third place was the article by Vedam S in collaboration with Keall PJ, published in 2003 (Vedam et al., 2003). They found that it was feasible to calculate respiratory movements from external respiratory signals by a four-dimensional computer. This provided theoretical support for the technical development of the later 4D-CT and also promoted the respiratory gating technology. At the top of the list of cited references is the article by McClelland et al. (2013), who published a review of respiratory motion models in 2013. They proposed that respiratory motion problems were a serious obstacle to the development of techniques for acquiring images or interventions, and that motion models offer a possible solution to these problems. For example, the gas exchange model of the lung was used to model the process of external respiration and study the mechanism and law of gas exchange in the alveoli. Respiratory movement control models use models to study control assumptions, such as the control of inspiratory time, expiratory time, and tidal volume. Additionally, 4D CT made it possible to study respiratory-induced organ motion, and Ehrhardt et al. (2011). generate a mean lung motion model based on 4D-CT data of the chest of different patients to extend the motion modeling capability. Furthermore, to establish an accurate assessment framework for objective assessment of deformable image registration (DIR), Castillo et al. (2009) validated the feasibility of landmark sets by manually generating large samples (>1,100) of corresponding lung landmark features from 4D-CT data of treatment plans and suggested practical strategies for routine clinical quality assurance of DIR spatial accuracy. In order to reduce the interference of respiratory movements during radiation therapy, Boda-Heggemann et al. (2016) proposed methods for establishing deep inspiration breath hold (DIBH). DIBH gating is a precise, reliable technique that is suitable for most patients and, with the advent of rapid dosing techniques, no longer leads to excessive treatment times. It improves the accuracy of dosimetry by expanding the treatment window and moving target of particle therapy, which is beneficial for complex treatment plans with steep dose gradients.

**FIGURE 8:**
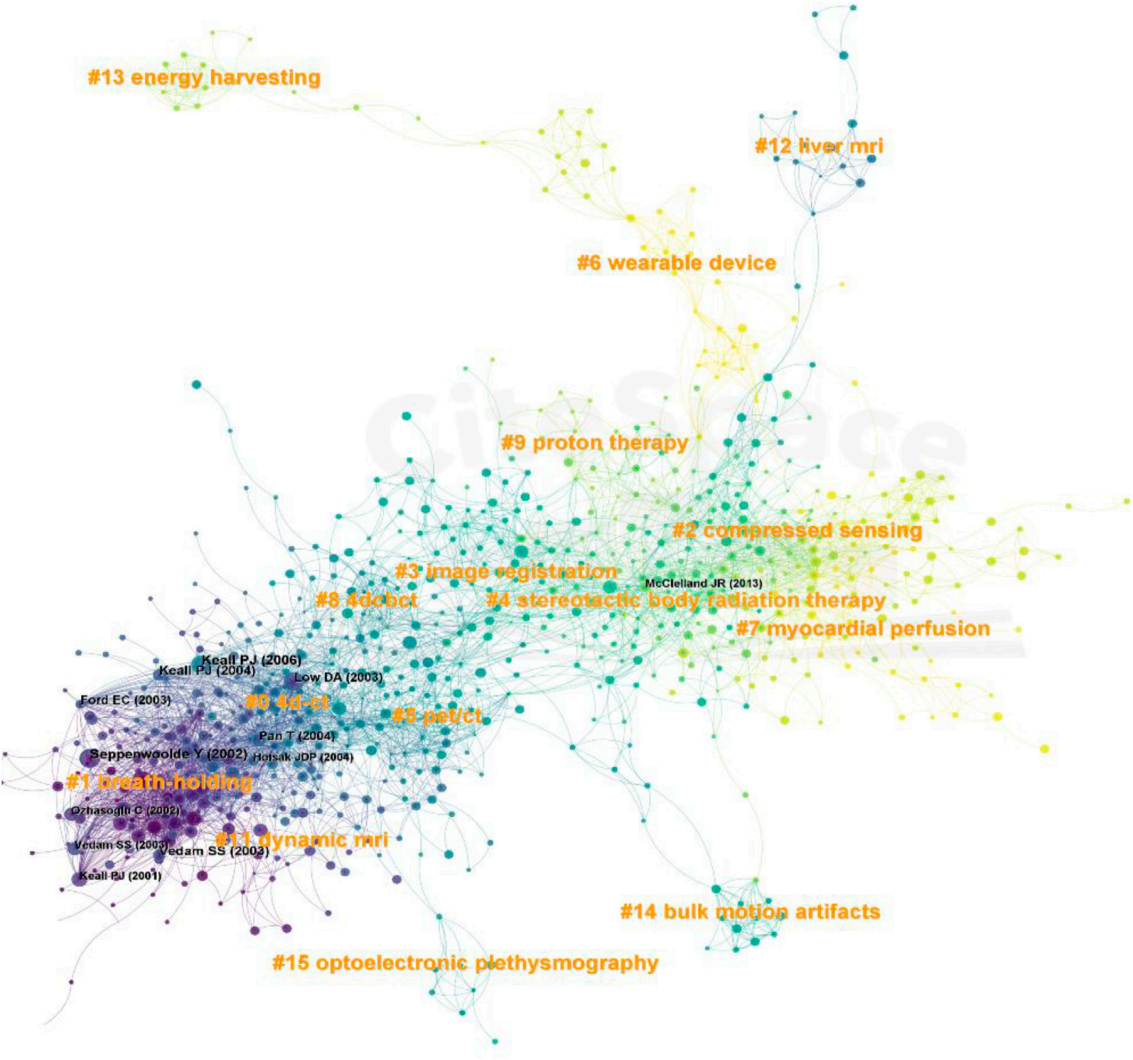
The clustering map of cited references related to respiratory biomechanics.

We conducted a cluster analysis of the cited references to examine the thematic and temporal distribution of these cited references (Figure 8). The Q-value in this figure was 0.7115 and the S-value was 0.8875, indicating good clustering and high reliability. It can be seen from the figure that the most recent cited topics are focused on imaging technology, proton therapy, optoelectronic plethysmography and wearable device. This hinted at a greater focus on wearables and imaging technology in recent years. Optoelectronic plethysmography (OEP), an accurate and proven method of measuring lung volume and chest wall motion, emerged in the 90s, and has since been used in an increasing number of respiratory biomechanics studies to evaluate respiratory patterns and asynchrony of respiratory strategies in patients various pathologies (Massaroni et al., 2017). Nowadays, OEP is an appropriate alternative method for monitoring and analyzing breathing patterns in children, adults and patients with respiratory diseases. In the future, OEP may help understand breathing patterns in respiratory diseases that are difficult to diagnose objectively, such as respiratory dysfunction, and may help improve treatment strategies. Regarding equipment, Charlton et al. (2018) published a review in 2018 on breathing rate (BR) estimation from the electrocardiogram and photoplethysmogram, outlining and evaluating algorithms for measuring BR, a key vital sign in the clinic. They suggested possible future directions for applications such as clinical monitoring, exercise monitoring, telemonitoring in the Home and remote video monitoring. Alizadeh, M et al. (Alizadeh et al., 2019) proposed an simpler and more comfortable approach for remote monitoring using a frequency modulated continuous wave (FMCW) radar, but they found that the accuracy of heart rate and respiration estimates with radar was 80%, compared to 94% for wearable devices. To improve the accuracy of the wearable device to measure BR, Al-Halhouli et al. (2019) developed a new stretchable sensor in 2019 based on silver nanoparticles that was low cost and easy to use.

### 3.7 Analysis of keywords

**FIGURE 9:**
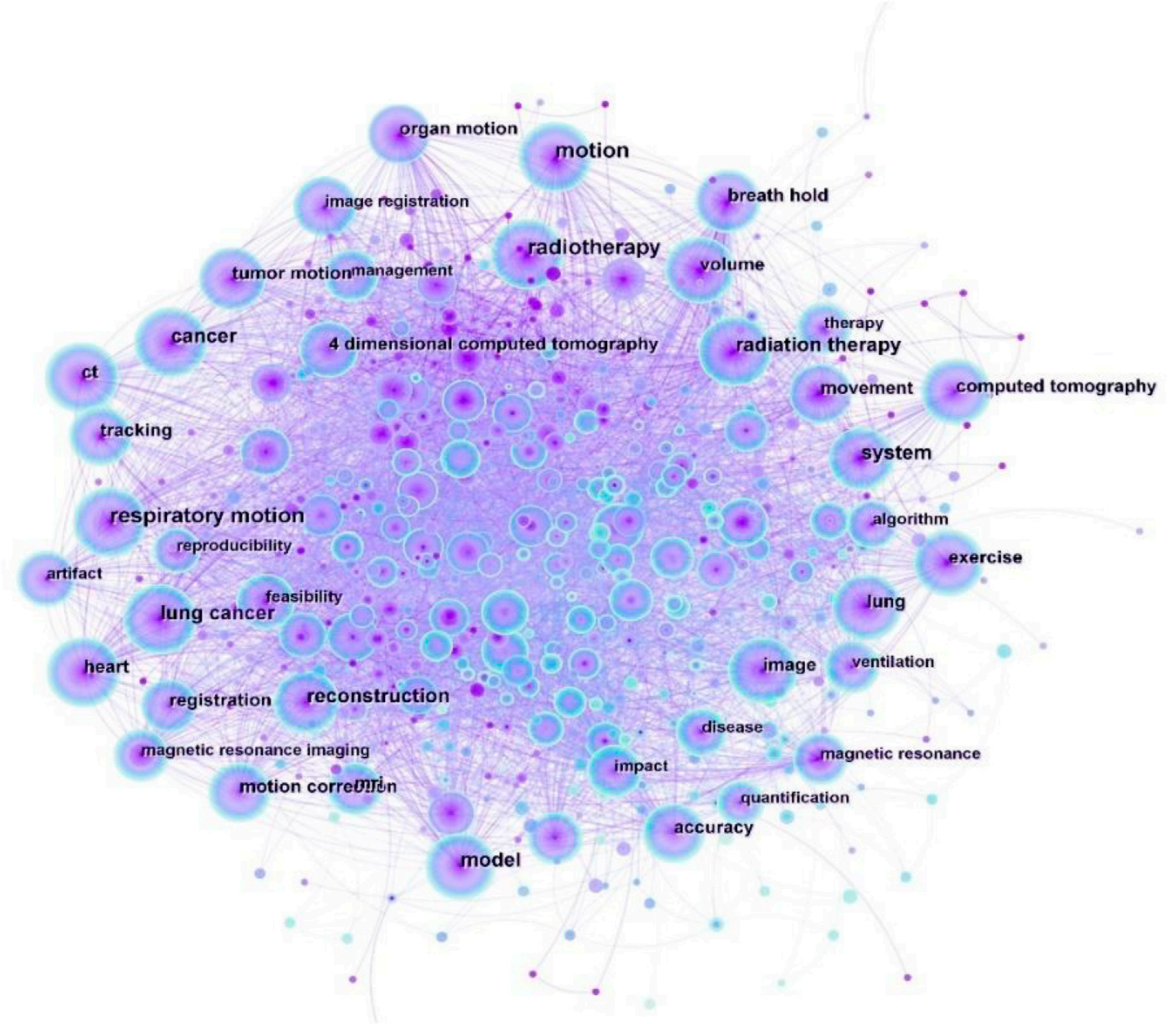
Map of keywords to respiratory biomechanics from 2003 to 2022.

An increase in the frequency of keywords or the number of cited keyword bursts over a certain period is believed to reflect cutting-edge themes or emerging trends (Liang et al., 2017). In the field of respiratory biomechanics, we found 609 keywords (Figure 9) that reveal the most popular topics. Table 6 is sorted by two indicators: frequency of keyword occurrence and centrality, respectively. However, high-frequency hot words are not necessarily positively correlated with their centrality, so they cannot be used as directional indicators for pinpointing research hot spots. On the other hand, keywords with high centrality (>0.1) are usually regarded as the critical points of keyword network mapping, which can reflect the current hotspots in the field. Overall, the top keywords were “radiotherapy,” “volume,” and “ventilation”.

**TABLE 6:** Top 10 frequency and centrality of keywords to respiratory biomechanics.

Rank	Keyword	Frequency	Rank	Keyword	Centrality
1	motion	359	1	volume	0.07
2	respiratory motion	341	2	movement	0.07
3	radiotherapy	301	3	ventilation	0.07
4	radiation therapy	223	4	breath hold	0.06
5	reconstruction	175	5	flow	0.06
6	lung cancer	166	6	breathing pattern	0.06
7	system	160	7	motion	0.05
8	cancer	158	8	model	0.05
9	model	153	9	heart	0.05
10	ct	148	10	exercise	0.05

In recent decades, advances in imaging technology have provided an increasingly wide range of potential applications for medical imaging, including diagnosis, treatment planning, and image-guided interventions. However, the issue of respiratory-induced organ motion remained a limiting factor. During image acquisition, it could lead to pseudo-images in the acquired images (Nehmeh and Erdi, 2008; Scott et al., 2009); and during interventions for image guidance, it limited the accuracy of the guidance (Hawkes et al., 2005). A number of approaches have been proposed to address the respiratory motion problem. The simplest method was to hold your breath, but this limited the acquisition or intervention time. To increase the time, respiratory gating appeared, but it had a limited window (e.g., end of expiration). Another solution was motion tracking. But the implantation can be invasive, and motion information is only available on the marker, not the entire region of interest (McClelland et al., 2013). Researchers were constantly updating their techniques for this purpose. Wijenayake and Park (2017) performed motion tracking by an RGB-D camera, which does not interfere with the natural breathing of the target and provides real-time depth information of the target surface. However, this method is suitable for static acquisition, while dynamic acquisition requires the use of OEP.

When it came to ventilation, mechanical ventilation, a common life-support technique, is used to treat respiratory failure or other clinical conditions requiring respiratory support by altering airway pressure to provide applied respiratory power. Respiratory diseases cause decreased lung function, dyspnea, cough, and chest pain, abnormal breathing patterns, as well as abnormal ventilation. Acute respiratory distress syndrome (ARDS) is a representative disease of abnormal lung oxygenation. A large number of alveolar collapses lead to the reduction of end-expiratory lung gas volume and lung compliance. Chronic obstructive pulmonary disease (COPD), a disease characterized by airflow limitation, has increased airway resistance and decreased lung elastic retraction, and increased lung volume will further reduce inspiratory muscle function and increase dyspnea (Kerti et al., 2018).

Appropriate use of mechanical ventilation can improve pulmonary ventilation and oxygenation and reduce dyspnea and respiratory muscle burden. However, inappropriate application may also lead to human-machine dyssynchrony, aggravating respiratory distress, which in turn may cause ventilator-associated lung injury, etc. Moreover, computational fluid dynamics (CFD) development has proven to be an important tool for assessing airflow and particulate deposition in human airways. Drug delivery is closely related to CFD. The main goal of drug delivery research is to indicate the optimal particle size to target a specific location in the lung to achieve the desired therapeutic effect. CFD simulation can simulate the lung inhalation process and visualize the key conditions of inhaled particles during respiration, helping doctors to evaluate the patient’s lung status and improve prognosis and treatment intervention, and also conducive to the development of required medical equipment (Francis and Saha, 2022). A review (Mutuku et al., 2020) describes aerosol deposition and clearance mechanisms, highlighting the role of bioaerosols in COVID-19 transmission, possible control strategies, and future challenges.

### 3.8 Research trends

**FIGURE 10:**
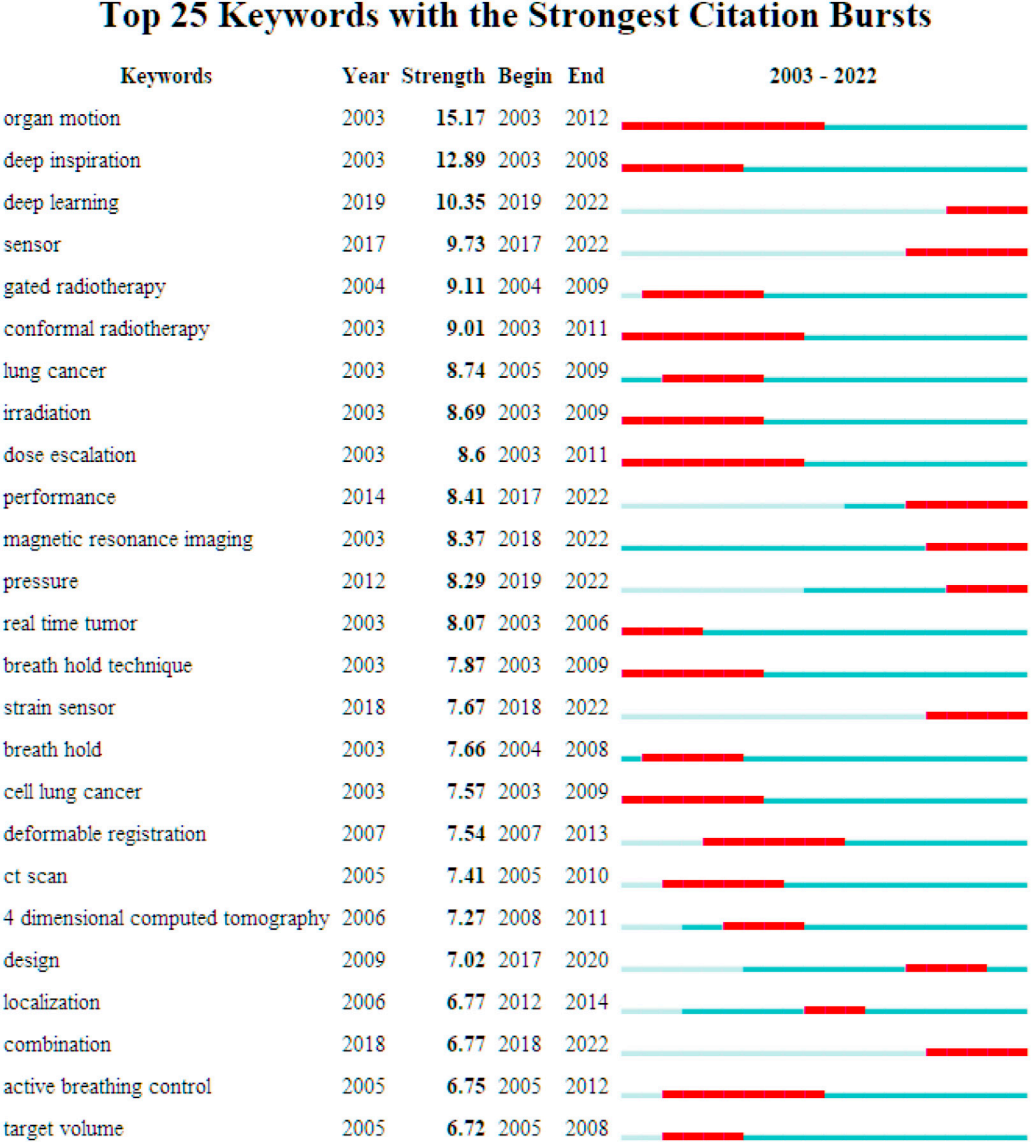
Top 25 keywords with the strongest citation burst.

Figure 10 shows the top 25 keywords with the most citation bursts from 2003 to 2022. Among these keywords, the most strength keywords were “organ motion,” “deep inspiration,” and “deep learning”. “Deep inspiration,” “deep learning,” “gated radiotherapy,” “conformal radiotherapy,” “irradiation,” “dose escalation,” “magnetic resonance imaging,” “breath hold technique,” “breath hold,” “CT scan,” “4-dimensional computed tomography,” “active breathing control,” “target volume” and “deformable registration” are interventions or diagnostic methods for remote respiratory biomechanics. “Organ motion,” “lung cancer,” “real time tumor,” “cell lung cancer” and “heart rate” are the objects of intervention for respiratory biomechanics. “Sensor, “strain sensor” and “positron emission tomography," are the equipment for respiratory biomechanics.

In recent decades, there has been an increasing incidence of malignant tumors, particularly the thoracic and abdominal tumors such as lung cancer and liver cancer, with high mortality rates. Lung cancer is one of the most common malignant tumors, and radiotherapy is the main treatment for patients with advanced lung cancer. The accuracy of target volume delineation directly affects the efficacy of radiotherapy in 3D or 4D radiotherapy, and the technology of precision radiotherapy is becoming more and more mature. Therefore, the precise delineation of target volume is crucial for precision radiotherapy. However, Lung cancer tumors move with breathing, leading to uncertainty in target coverage and dose delivery. Respiratory gated radiotherapy and deep inspiratory breath-holding are representative techniques (Park et al., 2022) that improve the accuracy of dose delivery by controlling tumor motion, but they need the patient’s cooperation. For those who cannot cooperate, continuous positive airway pressure (CPAP) can be used, which is less costly and provide lung and heart protection. Additionally, respiratory-gated PET-CT imaging (Kim et al., 2021) can be used to improve the quality of images and accurately calibrate the respiratory motion. If attenuation correction occurred problems, positron emission tomography (PET) can be affected. Now to address this issue, Pan, TS et al. developed an automated respiratory gating method, data-driven gated CT, which does not require external device gating (Pan et al., 2022).

The most recent burst keywords were “deep learning,” “sensor,” and “strain sensor”. Machine learning is a category of algorithms that focus on finding patterns in data and using those patterns to make predictions. Machine learning is part of the field of artificial intelligence and intersects with knowledge discovery and data mining. Supplementing CFD simulations with machine learning algorithms is becoming increasingly popular because this combination reduces the computational time of CFD simulations. Deep learning is a subfield of machine learning that utilizes artificial neural networks with multiple layers to analyze and extract features from large amount of data. Deep learning algorithms are general methods in solving complex problems with better performance. Shao, HC et al. (Shao et al., 2021) developed a deep-learning (DL)-based method to optimize the liver boundary DVFs after 2D-3D deformable registration to further improve the accuracy of subsequent biomechanical modeling and liver tumor localization. Now more machine learning methods are applied in the respiratory field to build optimization models so as to achieve a more accurate evaluation system.

At present, there are a variety of sensor technologies for breath detection, and here are some common sensor technologies: pressure sensors can measure changes in airflow pressure during breathing. They are commonly used in ventilators and other medical devices to monitor the respiratory flow and rhythm of patients. The chest strap sensor is fixed to the chest using a telescopic sensor band to measure the expansion and contraction of the chest. Respiratory rate, respiratory depth and respiratory rhythm can be evaluated by monitoring chest movement. Photoelectric sensors, such as infrared light sensors, can be used to measure the level of carbon dioxide in the breath. This sensor is often used to monitor respiratory ventilation, such as during anesthesia. Sound sensors can be used to monitor the sound patterns and frequencies produced during breathing. They can detect abnormal breathing patterns such as snoring or dyspnea. Flexible sensor is a kind of sensor with soft material and bending properties, which can be used to fit on the surface of the body. It can monitor the deformation of the chest or abdomen to measure respiratory movement. The research team (Jiang et al., 2023) designed a new type of humidity sensor using NaCl/halloysite nanotubes as materials. The sensor can detect changes in humidity during breathing and convert it into electricity. The sensor monitors the respiratory pattern by measuring the humidity in the respiratory gas, which provides data support for the evaluation of respiratory function. This humidity sensor provides a new method for respiratory monitoring and has a potential application prospect, providing energy solutions for self-powered medical devices and wearable technology. Besides, gas sensors are used to monitor and evaluate respiratory-related gas parameters. Such as volatile organic compounds (VOC) sensors (Di Gilio et al., 2020). VOC sensors can be used to detect volatile organic compounds in respiratory gases. These compounds can provide information about metabolic processes, lung disease and respiratory inflammation. Therefore, VOC sensor has potential application value in early diagnosis and monitoring of respiratory diseases. These sensor detection techniques can provide real-time and objective respiratory-related indicators, which can help medical staff to more accurately evaluate and monitor the respiratory condition of patients.

The 2019 Coronavirus disease, a severe acute respiratory syndrome infection, has spread rapidly around the world since its emergence in 2019 and has revolutionized our way of life. Patients recovering from COVID-19 may still face ongoing respiratory impairment caused by the virus and therefore require long-term post-discharge monitoring to closely assess pulmonary function during recovery (You et al., 2020). Therefore, the development of wearable sensors for respiratory biomechanical monitoring was very important for convenient home monitoring during rehabilitation and at the same time very promising for applications. Rehman et al. (2021) proposed a contactless small-scale movement monitoring system using software defined radio for detection of COVID-19 symptoms. Contactless technology for monitoring human movement and health working in a contactless manner may be one of the future directions. Now, more research is devoted to the development of self-powered multifunctional sensors that provide an efficient, low-cost, portable, and environmentally friendly means of active environmental assessment and personal biomonitoring (Xue et al., 2017; Su et al., 2020). For example, nanogenerator (Long et al., 2021) technology can be used to develop nano-sensors for breath detection. These sensors can be self-powered through the energy collection function of nanogenerator and can be used to detect and monitor respiratory parameters such as respiratory frequency, respiratory depth and respiratory patterns. This self-powered sensor provides real-time respiratory data without the need for an external power supply or frequent battery replacement.

**FIGURE 11:**
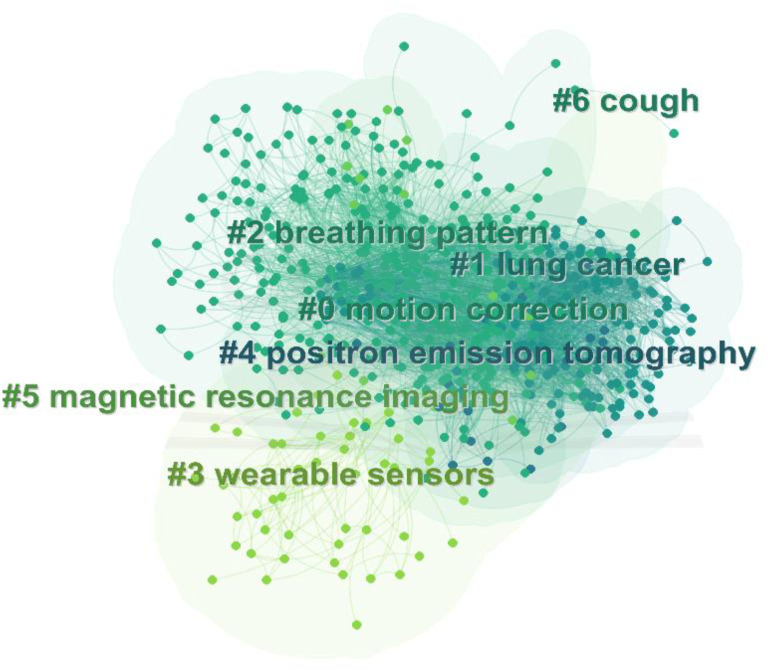
The clustering map of keywords related to respiratory biomechanics.

**FIGURE 12:**
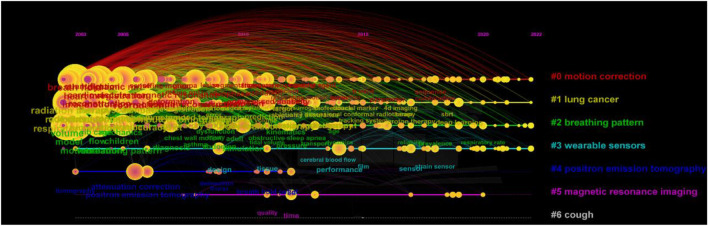
The timeline view of keywords related to respiratory biomechanics.

We performed a cluster analysis and applied it to the keywords related to respiratory biomechanics to provide a better understanding of current research topics. The Q value of 0.3934 and the S value of 0.7995 indicated that the clustering was appropriate and meaningful. A total of seven clusters were generated to reflect hot trends, and the most frequently occurring keywords were “motion correction,” “lung cancer,” “breathing pattern,” “wearable sensors,” “positron emission tomography,” “magnetic resonance imaging” and “cough” (Figure 11). In the timeline view (Figure 12), the warmer color were associated with the latest studies on “wearable sensors,” “positron emission tomography,” “magnetic resonance imaging” and “cough” were the latest studies, while “motion correction,” “lung cancer,” and “breathing pattern” were studied earlier. The citations of these hot topics may reflect the progress of medical technology and the importance of application in respiratory biomechanics research. The development of imaging technology provides a more comprehensive and accurate method for the detection and diagnosis of respiratory system. As a method of radiotherapy, proton therapy has been studied and concerned about the therapeutic effect of respiratory diseases. New technologies such as photoelectric plethysmography and wearable devices provide a more convenient and accurate tool for real-time monitoring and evaluation of respiratory biomechanics. At the same time, it may reflect the demand and concern for personalized medical care. Imaging technology, proton therapy and photoelectric plethysmography can provide individualized medical information and treatment options, which can help to better understand and manage respiratory diseases. The use of wearable devices can monitor individual respiratory status and health status in real time, and provide support for personalized health management.

## 4 Discussion

The study retrieved the core data of Web of Science using a search formula and obtained 2,850 respiratory biomechanics research papers published between 2003 and 2022. A visual analysis based on CiteSpace clarified the spatial and temporal distribution of respiratory biomechanics and research hotspots. Relevant publications have increased rapidly over the past 20 years with the highest values reached in 2019 (226), which may be related to the concern for respiratory biomechanics during the COVID-19 pendemic. New advances have also been made in respiratory diseases, respiratory monitoring techniques, respiratory rehabilitation and training, and the relationship between respiration and sleep. These studies provided important scientific support for the improvement of respiratory health, disease management and rehabilitation. Medical Physics published the most articles (263) and had the most citations (1,108) in this study. The average IF, Eigenfactor, CiteScore, SNIP, and SJR of cited journals were 6.01, 0.0395, 9.2, 1.777, and 1.704, respectively. It showed that there were more new methods and progress in the diagnosis and treatment of respiratory diseases by using medical physical techniques and methods in the past. Countries and institutions that published studies on respiratory biomechanics had relatively close cooperation. North America and Europe were pioneers in respiratory biomechanics research in the world, but an important trend was the boom of research results in Asian countries after 2019, with China being the most prolific country in the region. Close cooperation between countries and institutions helps to strengthen the integration and sharing of research resources, promote scientific exchanges and cooperation, and thus promote the progress and development of respiratory biomechanics research. North America and Europe are the pioneers of research, and their cooperation with Asian countries can bring more innovation and breakthroughs. China’s investment in scientific research and the improvement of the scientific research environment have also provided support for the explosive growth of respiratory biomechanics research. Of the 794 authors from different countries, KEALL PJ was the most productive author and MCCLELLAND JR was the most frequently cited author. The most frequently cited article was published by Keall PJ in 2006, on the report of respiratory movements, and the McClelland JR’s article in 2013 was the most representative and symbolic references. Their research promotes the development of radiotherapy and imaging of respiratory system, including respiratory gating technology, respiratory compensation and respiratory guided radiotherapy, and provides important scientific support for imaging evaluation and diagnosis of respiratory system. Cluster analysis of cited references showed that “imaging technology,” “proton therapy,” “optoelectronic plethysmography” and “wearable device,” were the most recently cited hot topics. The citations of these hot topics reflect the Frontier expansion of respiratory biomechanics research and the need for personalized medicine. Researchers are interested in emerging technologies and methods to explore and solve respiratory-related problems. Respiratory biomechanics research is more and more inclined to integrate and analyze a variety of data sources. This includes the combination of medical imaging, biological signals, clinical data and genomic information to fully understand the biomechanical properties of the respiratory system and the mechanism of the disease. The fusion of multimodal data can provide more information and help to reveal the complexity and individual differences of respiratory diseases.

From the key words analysis, “radiotherapy” has been a prominent and extensively researched topic in the field of respiratory biomechanics, which “organ motion,” “deep inspiration,” and “deep learning” as top Frontier keywords. Imaging techniques have been a major focus in addressing respiratory motion issues that impede image-guided innervations in radiotherapy. Biomechanical models of respiratory motion not only enhance medical imaging techniques and lung physiology, but also lead to the development of more accurate instrumentation with improved predictive health capabilities (McClelland et al., 2013; Maghsoudi-Ganjeh et al., 2021). The most recent burst keywords were “deep learning,” “ sensor,” and “strain sensor”. These sensor technologies are widely used in the fields of clinical medicine, health monitoring and sleep assessment, and provide real-time and accurate respiratory monitoring data for medical staff. With the progress of technology, more innovative breath detection sensor technology may emerge, including improving the accuracy, stability and wearability of the sensor. In the future, higher quality clinical trials and systematic reviews are crucial to further advance research in this significant area.

## 5 Limitation

There were several limitations to note about this study. Firstly, the data was not perfect, and we imposed restrictions on language and literature type, which may have limited the scope of our analysis. Further database supplements may be needed for the subsequent analysis. Secondly, while we carefully selected search terms and manual screened the literature, we could not guarantee that every piece of literature was completely relevant to the topic, nor can we be sure that we retrieved all literature related to the topic. Finally, the keywords in the query may not fully reflect the content of respiratory biomechanics, and it may be worthwhile to include other keywords, such as sensor and fluctuation analysis. Nevertheless, we believed that this study provides a useful description of the general situation and trends in the field from 2003 to 2022 and offers valuable recommendations for further research.

## 6 Conclusion

In summary, we used the visualization software CiteSpace to explore the development profile of respiratory biomechanics research. We have summarized important information about high-yielding authors, leading institutions, research areas, and hotspots of respiratory biomechanics research. Respiratory biomechanics research has been mainly conducted in developed countries, and collaborative networks have been formed among authors. The rapid development of respiratory biomechanics reflects the trend of globalization of scientific research. The cooperation and exchanges between different countries and regions have accelerated the dissemination and application of research results and helped to solve respiratory health problems around the world. Currently, the main research area of respiratory biomechanics is respiratory motion, and modeling of respiratory motion is becoming increasingly important for various applications in medical imaging. Respiratory biomechanics research is paying more and more attention to the development of individualized medical treatment and accurate diagnosis and treatment. By combining medical imaging, biomechanical simulations and individualized data, researchers can provide customized treatment options for each patient. This individualized method can better meet the specific needs of patients and improve the therapeutic effect and prognosis. As dyspnea is a common sequela of COVID-19, future research may turn to respiratory support techniques and respiratory monitoring techniques. With the advancement of mobile technology, more and more mobile devices and wearable devices have the function of collecting respiratory signals. For example, smart watches, smart phones, and smart health monitoring devices, etc., can collect respiratory movement, respiratory frequency and other data through built-in sensors, and perform fluctuation analysis. Now wearable sensors provide a way to passively capture daily movements; thus, respiratory monitoring devices will be further developed in the future to enable people to monitor their health at home. Additionally, the combination of respiratory biomechanics and non-contact detection technology is also a major highlight. Non-contact detection technology can obtain respiratory-related information without contact or minimizing contact, so as to avoid discomfort or interference that may be caused by sensor contact with the body. Compared with the traditional contact sensor, the non-contact technology has the advantages of real-time and convenience. This means that respiratory monitoring can be done more easily in daily life or in a medical environment without too much equipment or operation. We believe that respiratory biomechanics will continue to be studied and promoted in the future.

## Data Availability

The original contributions presented in the study are included in the article/Supplementary material, further inquiries can be directed to the corresponding authors.

## References

[B1] Al-Halhouli A. Al-Ghussain L. El Bouri S. Liu H. Zheng D. (2019). Fabrication and evaluation of a novel non-invasive stretchable and wearable respiratory rate sensor based on silver nanoparticles using inkjet printing technology. Polymers 11 (9), 1518. 10.3390/polym11091518 31540494 PMC6781180

[B2] Alizadeh M. Shaker G. Martins De Almeida J. C. Morita P. P. Safavi-Naeini S. (2019). Remote monitoring of human vital signs using mm-wave FMCW radar. IEEE Access 7, 54958–54968. 10.1109/access.2019.2912956

[B3] Blomberg B. Mohn K. G-I. Brokstad K. A. Zhou F. Linchausen D. W. Hansen B-A. (2021). Long COVID in a prospective cohort of home-isolated patients. Nat. Med. 27 (9), 1607–1613. 10.1038/s41591-021-01433-3 34163090 PMC8440190

[B4] Boda-Heggemann J. Knopf A-C. Simeonova-Chergou A. Wertz H. Stieler F. Jahnke A. (2016). Deep inspiration breath hold-based radiation therapy: A clinical review. Int. J. Radiat. Oncol. Biol. Phys. 94 (3), 478–492. 10.1016/j.ijrobp.2015.11.049 26867877

[B5] Castillo R. Castillo E. Guerra R. Johnson V. E. McPhail T. Garg A. K. (2009). A framework for evaluation of deformable image registration spatial accuracy using large landmark point sets. Phys. Med. And Biol. 54 (7), 1849–1870. 10.1088/0031-9155/54/7/001 19265208

[B6] Charlton P. H. Birrenkott D. A. Bonnici T. Pimentel M. A. F. Johnson A. E. W. Alastruey J. (2018). Breathing rate estimation from the electrocardiogram and photoplethysmogram: A review. IEEE Rev. Biomed. Eng. 11, 2–20. 10.1109/RBME.2017.2763681 29990026 PMC7612521

[B7] Chen C. Chen Y. (2005). Searching for clinical evidence in CiteSpace. AMIA Annu. Symp. Proc. 2005, 121–125. 16779014 PMC1560638

[B8] Chen C. Song M. (2019). Visualizing a field of research: A methodology of systematic scientometric reviews. Plos One 14 (10), e0223994. 10.1371/journal.pone.0223994 31671124 PMC6822756

[B9] Di Gilio A. Palmisani J. Ventrella G. Facchini L. Catino A. Varesano N. (2020). Breath analysis: Comparison among methodological approaches for breath sampling. Molecules 25 (24), 5823. 10.3390/molecules25245823 33321824 PMC7763204

[B10] Duan Z. H. Jiang Y. D. Tai H. L. (2021). Recent advances in humidity sensors for human body related humidity detection. J. Mater. Chem. C 9 (42), 14963–14980. 10.1039/d1tc04180k

[B11] Ehrhardt J. Werner R. Schmidt-Richberg A. Handels H. (2011). Statistical modeling of 4D respiratory lung motion using diffeomorphic image registration. IEEE Trans. Med. Imaging 30 (2), 251–265. 10.1109/TMI.2010.2076299 20876013

[B12] Fajnzylber J. Regan J. Coxen K. Corry H. Wong C. Rosenthal A. (2020). SARS-CoV-2 viral load is associated with increased disease severity and mortality. Nat. Commun. 11 (1), 5493. 10.1038/s41467-020-19057-5 33127906 PMC7603483

[B13] Francis I. Saha S. C. (2022). Computational fluid dynamics and machine learning algorithms analysis of striking particle velocity magnitude, particle diameter, and impact time inside an acinar region of the human lung. Phys. Of Fluids 34 (10), 101904. 10.1063/5.0106594

[B14] Groff D. Sun A. Ssentongo A. E. Ba D. M. Parsons N. Poudel G. R. (2021). Short-term and long-term rates of postacute sequelae of SARS-CoV-2 infection A systematic review. Jama Netw. Open 4 (10), e2128568. 10.1001/jamanetworkopen.2021.28568 34643720 PMC8515212

[B15] Hanson H. M. Eiben B. McClelland J. R. van Herk M. Rowland B. C. (2021). Technical Note: Four-dimensional deformable digital phantom for MRI sequence development. Med. Phys. 48 (9), 5406–5413. 10.1002/mp.15036 34101858

[B16] Hawkes D. J. Barratt D. Blackall J. M. Chan C. Edwards P. J. Rhode K. (2005). Tissue deformation and shape models in image-guided interventions: A discussion paper. Med. Image Anal. 9 (2), 163–175. 10.1016/j.media.2004.11.007 15721231

[B17] Hilberman M. Schill J. P. Peters R. M. (1969). On-line digital analysis of respiratory mechanics and the automation of respirator control. J. Thorac. Cardiovasc. Surg. 58 (6), 821–828. 10.1016/s0022-5223(19)42529-4 5260724

[B18] Hilberman M. Stacy R. W. Peters R. M. (1972). A phase method of calculating respiratory mechanics using a digital computer. J. Appl. physiology 32 (4), 535–541. 10.1152/jappl.1972.32.4.535 5026506

[B19] Huang L. Yao Q. Gu X. Wang Q. Ren L. Wang Y. (2021). 1-year outcomes in hospital survivors with COVID-19: A longitudinal cohort study. Lancet 398 (10302), 747–758. 10.1016/S0140-6736(21)01755-4 34454673 PMC8389999

[B20] Jiang Y. W. Duan Z. H. Fan Z. X. Yao P. Yuan Z. Jiang Y. D. (2023). Power generation humidity sensor based on NaCl/halloysite nanotubes for respiratory patterns monitoring. Sensors Actuators B-Chemical 380, 133396. 10.1016/j.snb.2023.133396

[B21] Jin Y-H. Cai L. Cheng Z-S. Cheng H. Deng T. Fan Y-P. (2020). A rapid advice guideline for the diagnosis and treatment of 2019 novel coronavirus (2019-nCoV) infected pneumonia (standard version). Mil. Med. Res. 7 (1), 4. 10.1186/s40779-020-0233-6 32029004 PMC7003341

[B22] Jodat R. W. Horgan J. D. Lange R. L. (1966). Simulation of respiratory mechanics. Biophysical J. 6 (6), 773–785. 10.1016/S0006-3495(66)86694-8 PMC1368042 5972377

[B23] Kamalabadi M. Ghoorchian A. Derakhshandeh K. Gholyaf M. Ravan M. (2022). Design and fabrication of a gas sensor based on a polypyrrole/silver nanoparticle film for the detection of ammonia in exhaled breath of COVID-19 patients suffering from acute kidney injury. Anal. Chem. 94 (47), 16290–16298. 10.1021/acs.analchem.2c02760 36379619

[B24] Karbing D. S. Leonhardt S. Perchiazzi G. Bates J. H. T. (2022). What is new in respiratory monitoring? J. Clin. Monit. Comput. 36 (3), 599–607. 10.1007/s10877-022-00876-4 35552970 PMC9103600

[B25] Keall P. J. Mageras G. S. Balter J. M. Emery R. S. Forster K. M. Jiang S. B. (2006). The management of respiratory motion in radiation oncology report of AAPM Task Group 76. Med. Phys. 33 (10), 3874–3900. 10.1118/1.2349696 17089851

[B26] Kerti M. Balogh Z. Kelemen K. Varga J. T. (2018). The relationship between exercise capacity and different functional markers in pulmonary rehabilitation for COPD. Int. J. Chronic Obstr. Pulm. Dis. 13, 717–724. 10.2147/COPD.S153525 PMC5836697 29535512

[B27] Kim J-S. Park C-R. Yoon S-H. Lee J-A. Kim T-Y. Yang H-J. (2021). Improvement of image quality using amplitude-based respiratory gating in PET-computed tomography scanning. Nucl. Med. Commun. 42 (5), 553–565. 10.1097/MNM.0000000000001368 33625179

[B28] Liang Y-D. Li Y. Zhao J. Wang X-Y. Zhu H-Z. Chen X-H. (2017). Study of acupuncture for low back pain in recent 20 years: A bibliometric analysis via CiteSpace. J. Pain Res. 10, 951–964. 10.2147/JPR.S132808 28479858 PMC5411170

[B29] Long Y. Li J. Yang F. Wang J. Y. Wang X. D. (2021). Wearable and implantable electroceuticals for therapeutic electrostimulations. Adv. Sci. 8 (8), 2004023. 10.1002/advs.202004023 PMC8061371 33898184

[B30] Maghsoudi-Ganjeh M. Mariano C. A. Sattari S. Arora H. Eskandari M. (2021). Developing a lung model in the age of COVID-19: A digital image correlation and inverse finite element analysis framework. Front. Bioeng. And Biotechnol. 9, 684778. 10.3389/fbioe.2021.684778 PMC8576180 34765590

[B31] Manber R. Thielemans K. Hutton B. F. Wan S. McClelland J. Barnes A. (2016). Joint PET-MR respiratory motion models for clinical PET motion correction. Phys. Med. And Biol. 61 (17), 6515–6530. 10.1088/0031-9155/61/17/6515 27524409

[B32] Massaroni C. Carraro E. Vianello A. Miccinilli S. Morrone M. Levai I. K. (2017). Optoelectronic plethysmography in clinical practice and research: A review. Respiration 93 (5), 339–354. 10.1159/000462916 28329750

[B33] McClelland J. R. Hawkes D. J. Schaeffter T. King A. P. (2013). Respiratory motion models: A review. Med. Image Anal. 17 (1), 19–42. 10.1016/j.media.2012.09.005 23123330

[B34] Mutuku J. K. Hou W. C. Chen W. H. (2020). An overview of experiments and numerical simulations on airflow and aerosols deposition in human airways and the role of bioaerosol motion in COVID-19 transmission. Aerosol And Air Qual. Res. 20 (6), 1172–1196. 10.4209/aaqr.2020.04.0185

[B35] Neelakantan S. Xin Y. Gaver D. P. Cereda M. Rizi R. Smith B. J. (2022). Computational lung modelling in respiratory medicine. J. Of R. Soc. Interface 19 (191), 20220062. 10.1098/rsif.2022.0062 35673857 PMC9174712

[B36] Nehmeh S. A. Erdi Y. E. (2008). Respiratory motion in positron emission tomography/computed tomography: A review. Seminars Nucl. Med. 38 (3), 167–176. 10.1053/j.semnuclmed.2008.01.002 18396177

[B37] Pan T. Thomas M. A. Luo D. (2022). Data-driven gated CT: An automated respiratory gating method to enable data-driven gated PET/CT. Med. Phys. 49 (6), 3597–3611. 10.1002/mp.15620 35324002 PMC9187617

[B38] Park J. Yea J. W. Oh S. A. Park J. Park J. W. Lee J. E. (2022). Efficacy and optimal pressure of continuous positive airway pressure in intensity-modulated radiotherapy for locally advanced lung cancer. Cancers 14 (17), 4308. 10.3390/cancers14174308 36077844 PMC9454671

[B39] Pollock S. Kipritidis J. Lee D. Bernatowicz K. Keall P. (2016). The impact of breathing guidance and prospective gating during thoracic 4DCT imaging: An XCAT study utilizing lung cancer patient motion. Phys. Med. And Biol. 61 (17), 6485–6501. 10.1088/0031-9155/61/17/6485 27523908

[B40] Rehman M. Shah R. A. Khan M. B. Abu Ali N. A. Alotaibi A. A. Althobaiti T. (2021). Contactless small-scale movement monitoring system using software defined radio for early diagnosis of COVID-19. IEEE Sensors J. 21 (15), 17180–17188. 10.1109/JSEN.2021.3077530 PMC8791440 35789227

[B41] Roldan-Valadez E. Yoselin Salazar-Ruiz S. Ibarra-Contreras R. Rios C. (2019). Current concepts on bibliometrics: A brief review about impact factor, eigenfactor score, CiteScore, SCImago journal rank, source-normalised impact per paper, H-index, and alternative metrics. Ir. J. Med. Sci. 188 (3), 939–951. 10.1007/s11845-018-1936-5 30511320

[B42] Ruan D. Fessler J. Balter J. Keall P. (2009a). TU-C-303A-03: Real-Time profiling of respiratory motion and its application to continuous horizon prediction. Med. Phys. 36 (6), 2724–2725. 10.1118/1.3182340

[B43] Ruan D. Fessler J. A. Balter J. M. Keall P. J. (2009b). Real-time profiling of respiratory motion: Baseline drift, frequency variation and fundamental pattern change. Phys. Med. And Biol. 54 (15), 4777–4792. 10.1088/0031-9155/54/15/009 19622852

[B44] Scott A. D. Keegan J. Firmin D. N. (2009). Motion in cardiovascular MR imaging. Radiology 250 (2), 331–351. 10.1148/radiol.2502071998 19188310

[B45] Seppenwoolde Y. Shirato H. Kitamura K. Shimizu S. van Herk M. Lebesque J. V. (2002). Precise and real-time measurement of 3D tumor motion in lung due to breathing and heartbeat, measured during radiotherapy. Int. J. Radiat. Oncol. Biol. Phys. 53 (4), 822–834. 10.1016/s0360-3016(02)02803-1 12095547

[B46] Shao H-C. Huang X. Folkert M. R. Wang J. Zhang Y. (2021). Automatic liver tumor localization using deep learning-based liver boundary motion estimation and biomechanical modeling (DL-Bio). Med. Phys. 48 (12), 7790–7805. 10.1002/mp.15275 34632589 PMC8678353

[B47] Su Y. Wang J. Wang B. Yang T. Yang B. Xie G. (2020). Alveolus-inspired active membrane sensors for self-powered wearable chemical sensing and breath analysis. Acs Nano 14 (5), 6067–6075. 10.1021/acsnano.0c01804 32271532

[B48] Vedam S. S. Keall P. J. Kini V. R. Mostafavi H. Shukla H. P. Mohan R. (2003). Acquiring a four-dimensional computed tomography dataset using an external respiratory signal. Phys. Med. And Biol. 48 (1), 45–62. 10.1088/0031-9155/48/1/304 12564500

[B49] Wang Y. Bao Y. Zhang L. Fan W. He H. Sun Z. W. (2013). Assessment of respiration-induced motion and its impact on treatment outcome for lung cancer. Biomed Res. Int. 2013, 872739. 10.1155/2013/872739 23862160 PMC3686059

[B50] Wijenayake U. Park S-Y. (2017). Real-time external respiratory motion measuring technique using an RGB-D camera and principal component analysis. Sensors 17 (8), 1840. 10.3390/s17081840 28792468 PMC5579577

[B51] Xu Q. Fang Y. Jing B. Q. Hu N. Lin K. Pan Y. (2021). A portable triboelectric spirometer for wireless pulmonary function monitoring. Biosens. Bioelectron. 187, 113329. 10.1016/j.bios.2021.113329 34020223 PMC8118703

[B52] Xue H. Yang Q. Wang D. Luo W. Wang W. Lin M. (2017). A wearable pyroelectric nanogenerator and self-powered breathing sensor. Nano Energy 38, 147–154. 10.1016/j.nanoen.2017.05.056

[B53] Yentes J. M. Liu W-Y. Zhang K. Markvicka E. Rennard S. I. (2022). Updated perspectives on the role of biomechanics in COPD: Considerations for the clinician. Int. J. Chronic Obstr. Pulm. Dis. 17, 2653–2675. 10.2147/COPD.S339195 PMC9585958 36274993

[B54] You J. Zhang L. Ni-jia-Ti M-y-d-l. Zhang J. Hu F. Chen L. (2020). Anormal pulmonary function and residual CT abnormalities in rehabilitating COVID-19 patients after discharge. J. Infect. 81 (2), E150–E152. 10.1016/j.jinf.2020.06.003 32512021 PMC7273134

[B55] Yuan Z. Zhao Q. N. Xie C. Y. Liang J. G. Duan X. H. Duan Z. H. (2022). Gold-loaded tellurium nanobelts gas sensor for ppt-level NO2 detection at room temperature. Sensors Actuators B-Chemical 355, 131300. 10.1016/j.snb.2021.131300

[B56] Zang X. Jiang Y. Chai Y. Li F. Ji J. Xue M. (2022). Tunable metallic-like transport in polypyrrole. Mater. Futur. 1 (1), 011001. 10.1088/2752-5724/ac44ab

[B57] Zheng J. Hou M. Liu L. Wang X. (2022). Knowledge structure and emerging trends of telerehabilitation in recent 20 Years: A bibliometric analysis via CiteSpace. Front. Public Health 10, 904855. 10.3389/fpubh.2022.904855 35795695 PMC9251196

